# Non-Canonical Amino Acids as Building Blocks for Peptidomimetics: Structure, Function, and Applications

**DOI:** 10.3390/biom13060981

**Published:** 2023-06-12

**Authors:** Tarsila G. Castro, Manuel Melle-Franco, Cristina E. A. Sousa, Artur Cavaco-Paulo, João C. Marcos

**Affiliations:** 1 CEB—Centre of Biological Engineering, University of Minho, Campus de Gualtar, 4710-057 Braga, Portugal; castro.tarsila@ceb.uminho.pt (T.G.C.); artur@deb.uminho.pt (A.C.-P.); 2 LABBELS—Associate Laboratory, Braga/Guimarães, Portugal; 3 CICECO—Aveiro Institute of Materials, Department of Chemistry, University of Aveiro, 3810-193 Aveiro, Portugal; manuelmelle@ua.pt; 4 BioMark Sensor Research—School of Engineering of the Polytechnic Institute of Porto, 4249-015 Porto, Portugal; criz.sousa38@gmail.com; 5 Centre of Chemistry, University of Minho, Campus de Gualtar, 4710-057 Braga, Portugal

**Keywords:** non-canonical amino acids, side-chain modifications, backbone modifications, peptidomimetics, foldamers, structure-function relationship

## Abstract

This review provides a fresh overview of non-canonical amino acids and their applications in the design of peptidomimetics. Non-canonical amino acids appear widely distributed in nature and are known to enhance the stability of specific secondary structures and/or biological function. Contrary to the ubiquitous DNA-encoded amino acids, the structure and function of these residues are not fully understood. Here, results from experimental and molecular modelling approaches are gathered to classify several classes of non-canonical amino acids according to their ability to induce specific secondary structures yielding different biological functions and improved stability. Regarding side-chain modifications, symmetrical and asymmetrical α,α-dialkyl glycines, Cα to Cα cyclized amino acids, proline analogues, β-substituted amino acids, and α,β-dehydro amino acids are some of the non-canonical representatives addressed. Backbone modifications were also examined, especially those that result in retro-inverso peptidomimetics and depsipeptides. All this knowledge has an important application in the field of peptidomimetics, which is in continuous progress and promises to deliver new biologically active molecules and new materials in the near future.

## 1. Introduction

This review focuses on the major differences between canonical and non-canonical amino acids, which give the latter the ability to be successfully incorporated into peptides, generating peptidomimetics for medical use and other applications [1,2]. To date, most of the findings about non-canonical amino acids (ncAA) derive from experimental studies. Driven by this fact, we also gather here predictions from molecular dynamics simulations concerning the structure and function relationship of these molecules, along with the most relevant results and applications from the experimental area. 

Peptides and proteins have been exhaustively studied over time, as they are vital molecules in most processes and body functions, providing the molecular machinery of life as we know it. In addition, these entities also play a plethora of fundamental functions, acting as hormones, neurotransmitters, inhibitors, etc., which are essential for human life [3,4,5]. However, the general use of proteins and peptides as therapeutic agents has major drawbacks in terms of bioavailability and biostability [6]. Degradation by proteases and problems concerning nonselective molecular receptors are some of the disadvantages of canonical peptide sequences [6,7,8]. In addition, the pharmacokinetics of peptides formed only by genetically encoded amino acids is also a process that does not favour the use of these molecules as drugs; common problems are poor oral availability, poor cell permeability, and rapid excretion through the liver and kidneys [9]. 

To overcome these problems, peptide-like molecules designed to mimic the function of natural peptides, called peptidomimetics, have been designed and tested [10,11,12]. Particularly, the enzymatic stability of a peptide is related to several factors such as amino acid composition, secondary structure, flexibility, and lipophilicity [13]. The most common and simple way to generate peptidomimetics is through modifications of native amino acids so that the new peptide shares a similar secondary structure but maintains or improves biological function. For instance, the hydrolysis of peptide bonds by proteases can be obstructed through the introduction of atypical moieties, such as d-amino acids, or by introducing an *N*-alkyl group [11,14,15,16]. 

The second type of peptidomimetics is based on more refined changes in (poly)peptide backbone, mainly by incorporating chemical foldamer moieties that will result in similar structural profiles or by modifications to the backbone. Recently, Lenci and Trabocchi reviewed peptidomimetics classes/types and classifications, which include chemical structures that drastically differ from the parent peptide but retain the scaffold, interactions, or function [9]. Here, the focus lies on amino acids and backbone modifications, preserving the peptide-like character. 

The rational design of new peptidomimetics is highly dependent on our knowledge of the structure-function relation of ncAA. In fact, very recent studies on the topic of peptidomimetics have reinforced the significance of ncAAs [17,18,19,20]. Do and Link, for instance, highlight their role with a focus on ribosomally synthesized and post-translationally modified peptides (RiPPs).

We aim to create and update an ncAA library, suggesting amino acid alternatives able to induce a specific secondary structure, i.e., with a foldamer profile, but also listing their applicability as building blocks. Secondly, we also present the peptidomimetics’ applicability in medicinal chemistry, listing some designed, tested, or approved peptides and pointing out the ncAA present. Lastly, we emphasize that computational tools have a great role in the design of peptidomimetics and are gaining relevance as predictive tools in peptide science [21,22,23,24]. 

### Amino Acids and Peptides: Structural Features and Properties

α-amino acids are organic molecules presenting a carboxylic (COOH) and an amine (NH_2_) group bonded to a common carbon atom, named alpha carbon (Cα). They are the fundamental building units of peptides and proteins. There are 20 natural amino acids encoded by the genetic code, widely recognized as the canonical amino acids, which constitute the most known proteins and enzymes [25].

**Figure 1:**
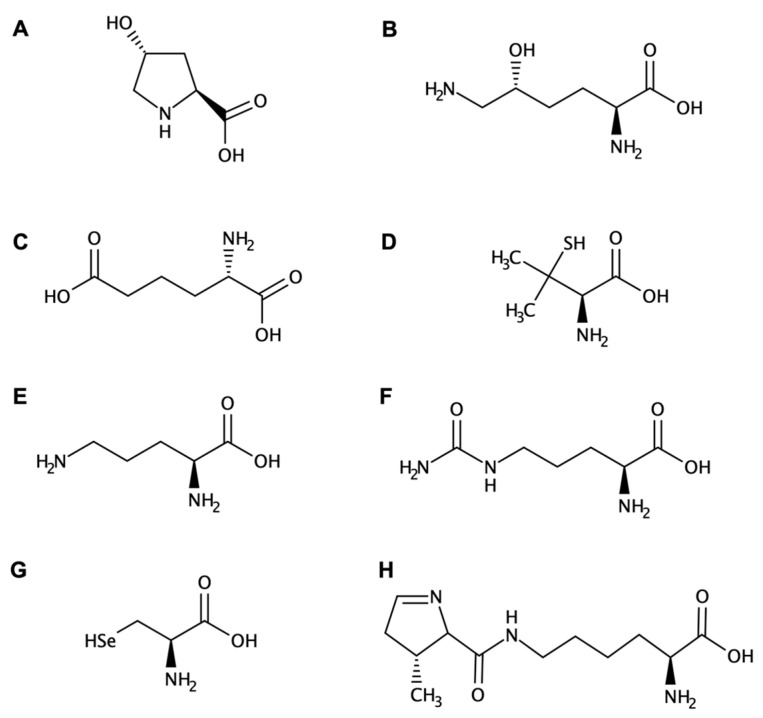
Two-dimensional structures of some non-canonical amino acids naturally found in nature or post-translational processes. (**A**) l-hydroxyproline, (**B**) hydroxylysine, (**C**) l-α-aminoadipic acid, (**D**) l-penicillamine, (**E**) l-ornithine, (**F**) citrulline, (**G**) selenocysteine, and (**H**) pyrrolysine.

Exceptions to the 20 canonical amino acids of natural occurrence are well known, some of them generated in post-translational processes and others found as free metabolites. For example, hydroxyproline (Figure 1A) and hydroxylysine (Figure 1B) occur on the protein collagen [26]. They are produced by hydroxylation of the amino acids proline and lysine, respectively, by the correspondent hydroxylase enzyme, as a post-translational modification [27,28]. The α-aminoadipic acid (Figure 1C) can be present in corn proteins or appears as a metabolite/intermediate in the lysine metabolism [29]. Likewise, penicillamine (Figure 1D) is an α-amino acid metabolite of penicillin, similar to cysteine, and it is used to treat arthritis [30]. Ornithine (Figure 1E) participates in the urea cycle as one of the products of the action of the enzyme arginase on l-arginine [31]. Citrulline (Figure 1F), naturally found in watermelon, is an amino acid derived from arginine [32]. 

Importantly, although by definition there are 20 l-amino acids encoded by the genetic code, there are two other residues that are proteinogenic: selenocysteine (Sec) and pyrrolysine (Pyl) (Figure 1G,H) [33,34]. Whereas Pyl appears only in proteins of Archaea organisms and a few bacterial genera [35], on the contrary, Sec is found in all kingdoms of life as the building block of selenoproteins. Sec, the 21st amino acid, is a cysteine (Cys) residue analogue with a selenol group in place of the thiol group and has already been found in 25 human selenoproteins and selenoenzymes [36]. 

Sec is encoded by a UGA codon, which is normally a stop codon but acts by performing a translational recoding; i.e., the mRNA reprograms the ribosome to read the message in alternative ways [37,38]. Selenium displays quite different properties compared to sulfur. It lowers Sec’s pKa and makes it a stronger nucleophile than Cys. While Pyl’s incorporation into polypeptides closely resembles the incorporation of canonical amino acids at sense codons, it occurs in response to an in-frame amber stop codon. Pyl participates in ribosomal translation because it is charged onto an amber suppressor tRNA_CUA_ by its own pyrrolysyl-tRNA synthetase [35].

Peptides are composed of chains of linked amino acid residues, which are classified as oligopeptides when short and are polypeptides when larger. The exact terminology, in accordance with the length, is quite variable. Some sources consider oligopeptide sequences of 2–10 amino acids, others consider sequences of 2–20, and in yet others, 2–40 residues are also reported [39]. Representative classes of oligopeptides are aeruginosins, cyanopeptolins, microcystins, microviridins, microginins, anabaenopeptins and cyclamides, which were divided based on their molecular structure and/or in the presence of specific moieties or amino acid derivatives [40,41]. Polypeptides are peptides that contain longer, continuous, and linear peptide chains. All proteins are polypeptides, but the reverse is not necessarily true, since a protein has a unique amino acid sequence encoded by a gene, which will fold in a specific way to perform a biological function.

**Figure 2:**
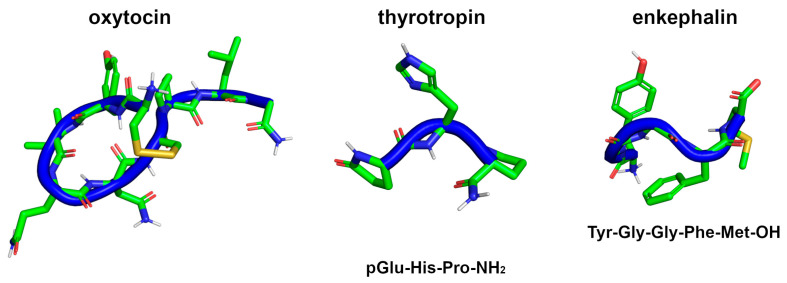
The 3D structures of the peptides, oxytocin (PDB ID: 7OFG), thyrotropin (PDB ID: 7X1U), and Met-enkephalin (PDB ID: 1PLW), with the respective sequences and backbone highlighted in blue ribbon.

Several naturally occurring peptides present, alone, important biological functions, being fully functional entities [39]. Relevant examples of peptides and small proteins are the vertebrate hormones, insulin (51 residues), glucagon (29 residues), and corticotropin (39 residues), and many drugs have been developed on their basis, including by using d-amino acids and other residue modifications [42]. Other examples of small naturally occurring peptides are the endogenous hormones oxytocin [43] and thyrotropin [44] and the neurotransmitter enkephalin [45], consisting of only 9, 3, and 5 amino acids, respectively (Figure 2). 

The biological function of a peptide is directly connected to the amino acid sequence and, very often, to the resulting secondary structure (SS). That is why understanding the role of an amino acid in the peptide structure and its interactions is the key to proposing a rational design of more stable and functional analogues. Modifications in the amino acid side chain or the peptide backbone can alter the normal configuration of φ and ψ, stabilizing a conformation or generating a new one. These dihedral angles are most important for peptide conformation and will be different for ncAA [46,47].

Remarkable examples where the amino acid content induces a specific SS that imparts function are the cell-penetrating peptides (CPP). This class comprises the family of antimicrobial peptides (AMP), which are membrane-active peptides. The CPPs present great potential as drug-delivery peptides, and the AMPs are promising antibiotic candidates [48]. Many other examples can be cited, and, importantly, all of them can be optimized with the insertion of ncAA to stabilize SS and enhance proteolytic stability.

## 2. Peptidomimetics Design

In order to analyze the main differences between canonical and non-canonical amino acids and peptidomimetics design, this review is divided into a compilation of what is known about amino acid side-chain modifications and then a review of the peptide backbone modifications.

### 2.1. Structural Properties of Non-Canonical Amino Acids

Non-canonical amino acids are organic molecules also containing an amine and a carboxylic acid group but are not directly encoded by the genetic code. Several residues are found in nature, and a large array can be synthesized [16,49]. 

**Figure 3:**
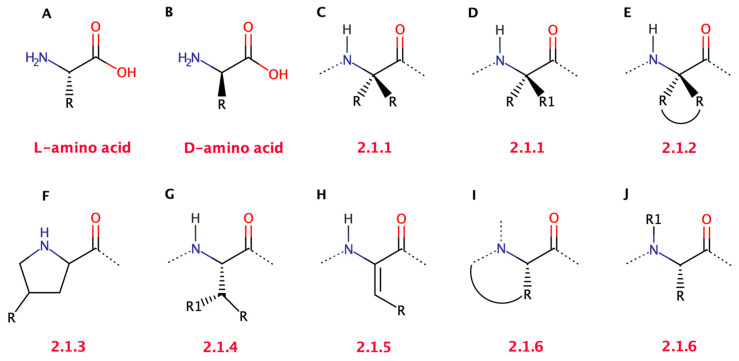
General structure of an encoded l-amino acid (**A**) and of its D form (**B**). Symmetrical α,α-dialkyl glycine (**C**), asymmetrical α,α-dialkyl glycine (**D**), cyclized amino acids (known as Ac_n_c) (**E**), proline analogues (**F**), β-substituted amino acid (**G**), general α,β-dehydroamino acid (**H**) with *N*-cyclization (**I**) and *N*-alkylation (**J**). Below the structure, the section where the class of amino acids is discussed is indicated.

The incorporation of ncAA into peptides is one of the approaches to generating peptidomimetics able to overcome the problems previously mentioned concerning the pharmacokinetics and enzymatic stability of natural peptides as drugs. In fact, the replacement of natural amino acids often results in higher activity and increased biological stability [6,10,14,50]. Figure 3 summarizes the most common natural and artificial modifications applied to encoded amino acids, used to generate peptidomimetics.

α,α-Dialkyl glycines, hydroxyproline, and α,β-dehydro amino acids are representatives naturally found in peptides of several organisms [51,52]. Nature shows us that animals and plants can fight against microbes using antimicrobial peptides [53,54]. Many of these peptide sequences carry ncAA, showing that it is possible to translate this defense mechanism from nature into the design, simulation, synthesis, and application of new peptidomimetics [11,12,14,55]. 

#### 2.1.1. Symmetrical and Asymmetrical α,α-Dialkyl Glycines 

**Figure 4:**
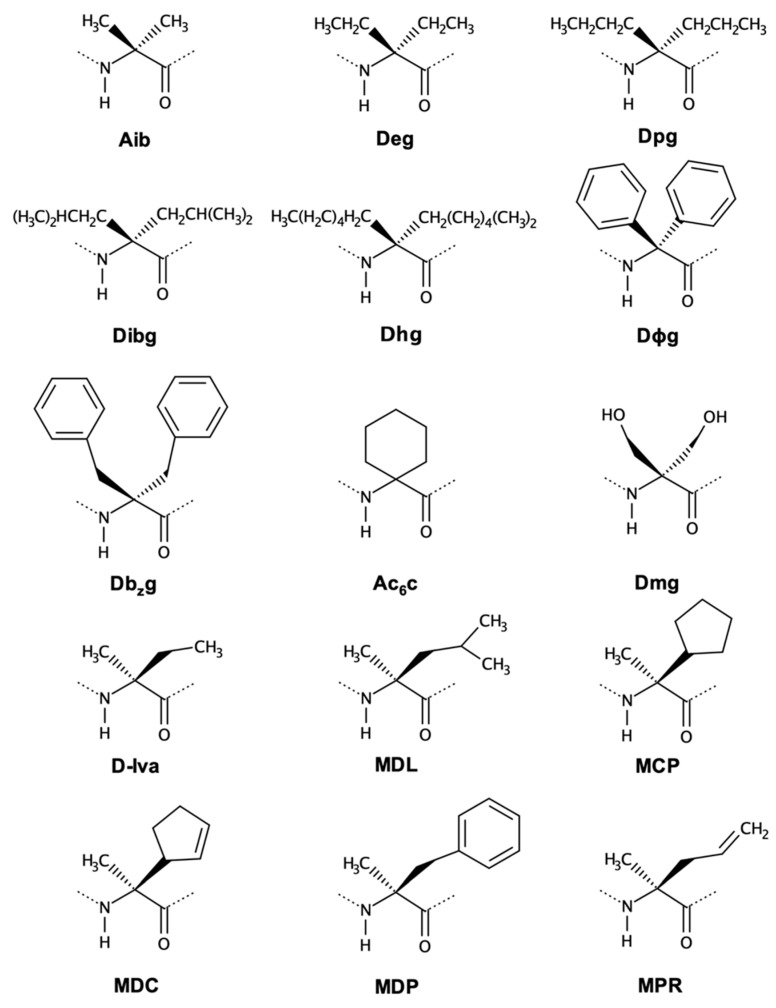
Two-dimensional structures of α,α-dialkyl glycines: α-amino isobutyric acid (Aib), α,α-diethyl glycine (Deg), α,α-dipropyl glycine (Dpg), α,α-di-isobutyl glycine (Dibg), α,α-dihexyl glycine (Dhg), α,α-diphenyl glycine (DΦg), α,α-dibenzyl glycine (Db_z_g), α,α-cyclohexyl glycine (Ac_6_c), and α,α-dihydroxymethyl glycine (Dmg). The asymmetrical d-α,α-dialkyl glycines investigated were d-Iva, MDL (α-methyl-d-leucine), MCP (2-amino-2-cyclopentylpropanoicacid), MDC (2-amino-2-(2-cyclopentenyl)propanoic acid), MDP (α-methyl-d-phenylalanine), and MPR (2-amino-2-methyl-4-pentenoic acid).

The most widely studied class of ncAA is probably the class of α,α-dialkyl glycines (Figure 4). This type of residue is found in many naturally occurring peptides, especially in antimicrobial peptides [56,57,58]. The Aib (α-aminoisobutyric acid) is the prototype of this class and is known to restrict the dihedral angles to generate α-helical conformations [49,59,60]. Figure 4 shows reported symmetric and asymmetric disubstituted glycines. 

Peptaibols are small–medium peptides that belong to the class of AMPs and have this name because they are rich in the ncAA Aib. Many peptaibols interact with cell membranes through a barrel-stave channel model. They are mostly helical entities, which allow the optimal channel formation necessary for biological function. We reported the structural properties of a series of ncAA amino acids inserted in different peptaibols: symmetrical α,α-dialkyl glycines for Peptaibolin and Alamethicin [61,62] and asymmetrical α,α-dialkyl glycines for Zervamicin II and Antiamoebin I [63]. 

The molecular dynamics simulations performed with this collection of ncAA indicate that some residues are more capable of inducing α-helical conformations and promoting spontaneous membrane permeation. In Peptaibolin, a Leu-Aib-based peptide, the substitution of native Aib for Dhg or Ac_6_c is capable of maintaining the ideal α-helical structure necessary for AMP function. However, all proposed peptidomimetics generated by symmetrical α,α-dialkyl glycines are able to successfully permeate a POPC (1-palmitoyl-2-oleoyl-sn-glycero-3-phosphocholine) membrane [61]. Experimentally, Peptaibolin analogues bearing Ac_6_c and Deg are the peptides with higher permeating ability, evidencing a correlation between the length and bulk of the α,α-dialkyl glycines side chain and the ability of the corresponding peptides to permeate the membranes [64].

In the case of Alamethicin, the bulky α,α-dialkyl glycines Dhg, Dϕg, and Db_z_g imposed more helical-constrained structures, and in a medium simulating a membrane environment, Deg, Ac_6_c, and Dhg were the amino acids that induced higher peptide helicity. 

Molecular dynamics simulations and free energy calculations of Alamethicin suggested an ideal peptide sequence based on the foldamer profile and energetics of each tested ncAA. This computational study resulted in an Alamethicin peptidomimetic with the following sequence: Ac-Dhg-Pro-Deg-Ala-Dhg-Ala-Gln-Dhg-Val-Aib-Gln-Leu-Dhg- Pro-Val-Dhg-Deg-Glu-Gln-Phe [62].

Symmetrical α,α-dialkyl glycines are achiral disubstituted amino acids; thus, dihedrals should be sampled that are typical of right-handed or left-handed helix configurations with similar probability. In contrast, a tendency for L configuration was observed, probably due to the encoded amino acids in the neighborhood, which influences the ncAA towards a similar structural arrangement.

Aib has been under investigation for many years. This ncAA was successfully incorporated in peptides such as enkephalin (replacing both Gly), bradykinin (replacing Phe), and angiotensin II (replacing Asp in position 1) [65,66,67,68,69], generating active and constrained peptidomimetics. Furthermore, Ac_6_c (1-aminocyclohexane-1-carboxylic acid) has been tested on enkephalin and endomorphin peptides to achieve peptidomimetics with large activity in vivo [70,71]. Ac_6_c is both an α,α-dialkyl glycine (because it is alkyl disubstituted at Cα) and a residue of Ac_n_c residues, in which the chains attached to the Cα are involved in a Cα to Cα cyclization.

Ross and co-workers reported in 1993 the synthesis of α-amino acids, including three asymmetrical α,α-dialkyl glycines [72]. Mendel and co-workers [49] reported protein biosynthesis with conformationally restricted residues, addressing different classes of amino acids, which included Iva and other asymmetrical disubstituted amino acids. This approach successfully generated peptides with well-defined secondary structures.

Recently, Das and co-workers [55] cited the symmetrical Deg, Dpg, Dibg, Dhg, DΦg, and Db_z_g as foldamers inspired by peptaibols. The success in the application of these amino acids can inspire the design of a great variety of symmetrical and asymmetric glycines, as well as their synthesis and screening through MD simulations. 

Peptaibol research is still a growing field, with synthetic peptides being designed for novel applications such as agrochemicals, as recently reported by Zotti and colleagues [73]. 

As for asymmetrical α,α-dialkyl glycines, they are chiral molecules and were simulated in D configuration, based on the d-Iva (isovaline) naturally present in the peptaibols Zervamicin and Antiamoebin. The d-amino acids studied induced the helical conformations required for the antibiotic function, but they importantly increase overall stability against proteolysis [63,74,75]. We highlight the residues MDL and MDP as the most promising helical inducers, regardless of the position in which they are inserted. 

#### 2.1.2. Cα to Cα Cyclized Amino Acids—Ac_n_c Residues

**Figure 5:**
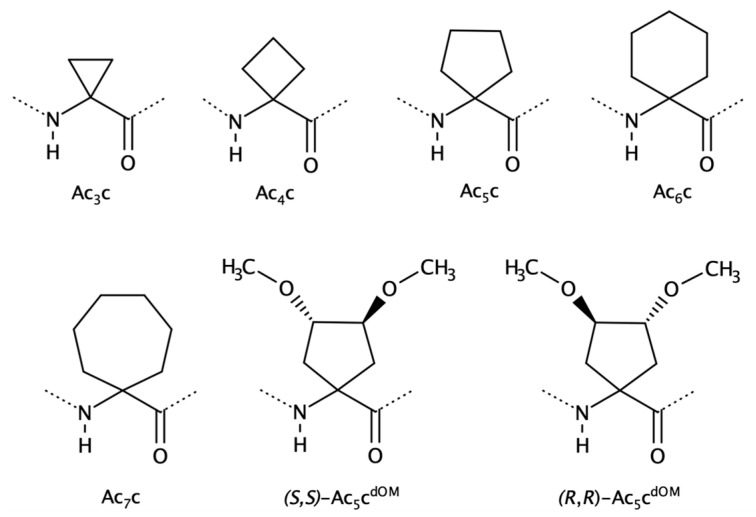
Two-dimensional structures of non-canonical Ac_n_c (1-aminocycloalkane-1-carboxylic acids) residues, where n refers to the size of the cycle: Ac_3_c, Ac_4_c, Ac_5_c, Ac_6_c, Ac_7_c, (*S*,*S*)-Ac_5_c^dOM^, and (*R*,*R*)-Ac_5_c^dOM^.

Cyclized Ac_n_c residues (Figure 5) have been widely studied over the past decades through experimental and computational methods [76,77,78,79,80,81,82,83]. The conformational preferences of these residues vary according to the cycle. 

Previous experimental and modelling findings indicate that Ac_n_c cycles with more than 3 atoms (n = 4–12) explore, mostly, the main chain geometry similar to Aib (*φ*, *ψ* ≈ ±60°, ±30°) which is typical of α-helix or 3_10_-helix SS [76,83,84,85,86,87,88]. The residues Ac_5_c (1-aminocyclopentane-1-carboxylic acid) and Ac_6_c (1-aminocyclohexane-1-carboxylic acid) have been found to yield γ-turn conformations in small peptides [78,89,90,91]. On the other hand, Ac_3_c (1-aminocyclopropane-1-carboxylic acid) is the only member of Ac_n_c family that prefers molecular geometries in the *bridge* region (*φ*, *ψ* ≈ ±90°, 0°), and this particularity has been the subject of several studies in recent decades [92,93,94,95,96].

This class of amino acids has been investigated to control secondary structures and generate new bioactive peptides [76,81]. The Ac_6_c residue has been incorporated, for instance, in helical AMP [62] or neurotransmitters [70]. 

Bulkier side chains, such as Ac_9_c, Ac_10_c, Ac_11_c, and Ac_12_c, have been frequently addressed in the past regarding their role as stronger helix formers [83,86,87,88] but also to increase peptide hydrophobicity to improve biological activity as agonists or antagonists towards a specific target [97]. 

#### 2.1.3. Proline Analogues

**Figure 6:**
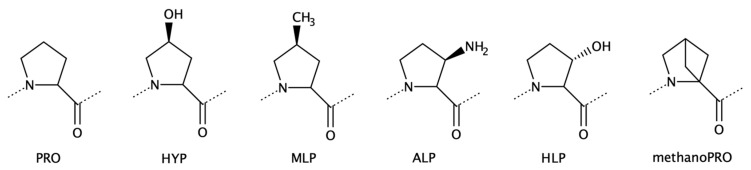
Two-dimensional structures of the encoded amino acid Pro and proline analogues. From left to right: l-Pro, *cis*-4-hydroxy-l-proline (Hyp), *cis*-4-methyl-l-proline (MLP), *cis*-3-amino-l-proline (ALP), *trans*-3-hydroxy-l-proline (HLP), and 2,4-methano-l-proline (methanoPRO).

Proline analogues (Figure 6) represent a class with unique conformational features, since the natural Pro residue is known to disrupt or prevent α-helix SS and favors the formation of β-turn structures. Amino acid analogues of proline have been studied experimentally and computationally to understand structure preference and applications [98,99,100,101,102]. Pro derivatives have been found in proteins of microbial and marine species [6]. 

We reported the incorporation of proline analogues into the peptaibols Antiamoebin I and Zervamicin II [63], which were studied by MD simulations. The ncAA *cis*-3-amino-l-proline (ALP) presented a foldamer profile, increasing the content of amino acids in α-helix in both peptaibols. These peptides naturally carry Hyp; therefore, we tested if another proline analogue would be able to increase the stability of helical conformations. The findings indicate that ALP and *trans*-3-hydroxy-l-proline (HLP) also act as good helical inducers.

Although proline is known to bend helical conformations, β-turn structures can be accommodated into helical backbones (with a hydrogen-bonding pattern i → i + 3) [98]. One proof of this is the structure of collagen, in which consecutive Pro and Hyp residues generate a helix [103].

#### 2.1.4. β-Substituted and Planar Amino Acids

**Figure 7:**
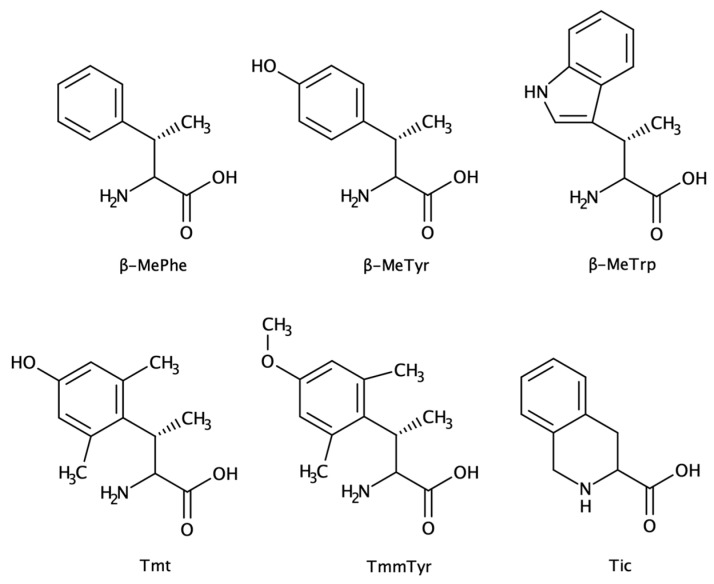
Structures of selected examples of non-canonical β-substituted amino acids. From left to right and top to bottom: β-MePhe, β-MeTyr, β-MeTrp, Tmt (trimethyltyrosine; β-methyl-2′,6′-dimethyltyrosine), TmmTyr (trimethyl-metoxytyrosine; β-methyl-2′,6′-dimethyl-4′metoxytyrosine), Tic (1,2,3,4-tetrahydroisoquinoline).

β-substituted amino acids (Figure 7) have been used to generate more potent peptidomimetics of naturally occurring peptide hormones, such as opioid peptides, angiotensin, or somatostatin [6,104]. Natural amino acids such as Phe, Trp, and Tyr are found in the pharmacophore of many peptide hormones. The addition of alkyl groups to the β position proved to be a powerful strategy to rigidify the residue, by constraining the rotation between Cα and Cβ, and to enhance the activity [11,105,106,107,108,109]. 

The insertion of this type of amino acid in peptidomimetics hormones should consider multiple factors, as the conformational state should match the target interaction partner (receptors, enzymes, membranes) [110]. The main goal will be to gain prolonged biological activity, due to the presence of an alkyl group [105]. Hruby and co-workers gathered inputs from pharmacology, computational chemistry, and biophysical analysis to better understand and apply this class of ncAA and found out that the Tic amino acid not only was able to maintain the β-turn related to the activity of somatostatin peptide but resulted in more potent and selective peptidomimetics for μ-opioid receptors [111]. 

#### 2.1.5. α,β-Dehydroamino Acids

**Figure 8:**
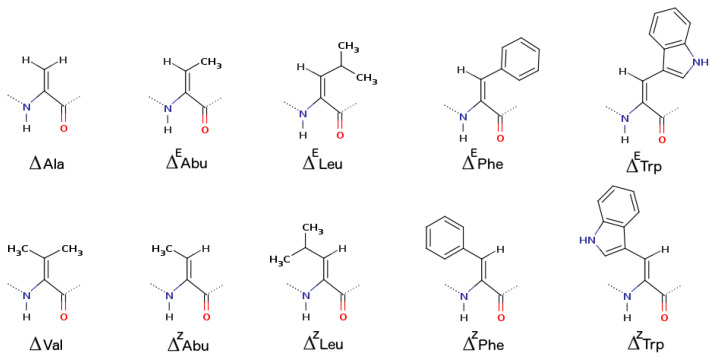
Two-dimensional structures of non-canonical α,β-dehydroamino acids: dehydroalanine (ΔAla), dehydrobutyrine (ΔAbu), dehydroleucine (ΔLeu), dehydrophenylalanine (ΔPhe), dehydrotryptophan (ΔTrp), and dehydrovaline (ΔVal). Those that present Z/E forms are ΔAbu, ΔLeu, ΔPhe, and ΔTrp.

α,β-dehydroamino acids (Figure 8) are ncAA amino acids naturally found in peptides [52,112,113]. The lack of asymmetry, due to the planar hybridization sp^2^ of the Cα carbon, structurally separates this class of amino acids from the canonical ones. In addition, these residues can present β-substituents, such as isomers Z and E, and the possibility of π-electron conjugation. All these properties contribute to a very specific constraint that influences the bioactivity and applications of these dehydropeptides.

The conformational properties of peptides carrying α,β-dehydroamino acids have been extensively reviewed in the past [112,113,114,115,116,117,118] but remain a hot topic today [119,120,121]. The residues dehydroalanine (ΔAla), dehydrobutyrine (ΔAbu), and dehydrophenylalanine (ΔPhe) are the most investigated [122,123,124,125,126,127,128]. ΔPhe has been intensively studied computationally [129,130]. 

This type of residue favors the formation of β-turns. In small peptides, when the dehydroamino acid is placed in the second position, especially ΔPhe, β or γ turns are the most probable arrangements. In intermediate or long peptides, sequential placement or sequential repeats of ΔPhe induce repeated β-turns that can be accommodated in a 3_10_-helix or even in an α-helix [131,132].

Applications for this class of amino acids were studied by us, combining experimental and computational approaches. In 2015, peptide hydrogelators carrying α,β-dehydroamino acids were evaluated computationally to assess their ability to self-assemble as a hydrogel [133]. This type of hydrogel can be used for drug delivery purposes. We proved that the aggregation process occurs due to the non-canonical ΔPhe, which interacts with the Npx (naproxen) group also present in our model peptides, through π–π interactions. We also investigated the affinity of dehydropeptides with αvβ3 integrin receptors using molecular docking methods [134]. Here, ΔPhe was inserted in the peptide construct Npx-l-Ala-Z-ΔPhe-Gly-Arg-Gly-Asp-Gly-OH, where the hydrogelator module Npx-l-Ala-Z-ΔPhe seems not to hinder the molecular recognition between RGD epitope and the αvβ3 integrin receptor. Thus, combining the hydrogelator module with other targeting epitopes is a feasible strategy for producing hydrogels with tailor-made cell specificity. Furthermore, recently, dipeptides carrying dehydroamino acids were addressed as new supergelators for drug delivery [119]. Again, the preferable interaction mode among the dipeptide units is π-stacking interactions. 

Regarding ΔAbu, *Elisidepsin* represents a synthetic, marine-derived peptide that is active in a wide variety of cancer cell types. This peptide is also a depsipeptide [135]. *Elisidepsin* is a derivative from the kahalalide family [136], i.e., a family of natural dehydro-aminobutyric acid-containing peptides.

#### 2.1.6. *N*-Cyclization and *N*-Alkylation

**Figure 9:**
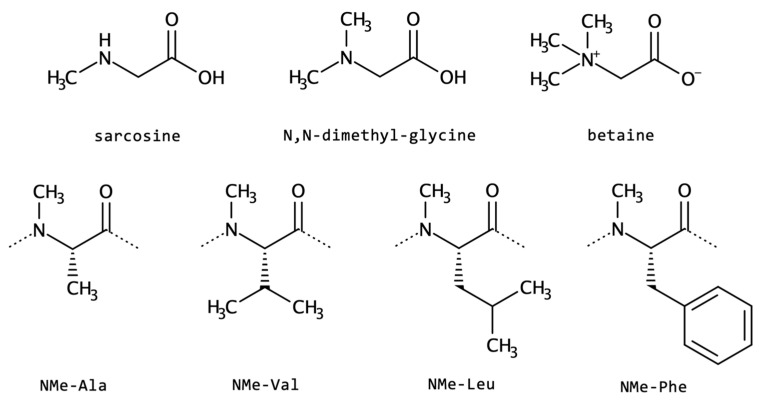
Two-dimensional structures of representative examples of *N*-alkylated non-canonical amino acids.

*N*-Alkylated-α-amino acids are widespread in nature. The most abundant representatives are the *N*-methyl forms. In fact, *N*-methyl-glycine, also known as sarcosine; *N*,*N*-dimethylglycine; and betaine are well-studied ncAA (Figure 9) that can be found as monomers, embedded into complex peptides or within non-peptide natural substances [137].

Another example, *N*-methyl-leucine, is present in the first position of the glycopeptide antibiotic vancomycin. This *N*-terminal is responsible for potency and binding to the peptidoglycan wall of Gram-positive bacteria [138,139]. The immunosuppressant *cyclosporine A* is rich in this same ncAA, but it also contains an *N*-methyl-valine. *Actinomycin D* is a chemotherapeutic drug that also contains *N*-methyl-valine and *N*-methyl-glycine. All of these three examples are depsipeptides, a class of peptidomimetics also presenting changes in the backbone and further discussed in Section 2.2. 

The industrial production of *N*-methyl-l-alanine or *N*-methylantranilate through fermentative routes has been established by using engineered *Corynebacterium glutamicum*. Recently, the metabolic engineering of *C. glutamicum* for de novo production of *N*-methyl-phenylalanine was described, based on the reductive methylamination of phenylpyruvate [140].

*N*-alkylated and *N*-cyclized ncAA affects the conformational flexibility and interaction pattern of the peptide backbone. The absence of the typical hydrogen bond donor NH disturbs the expected intramolecular hydrogen bonds, giving space to other arrangements and interactions. However, peptides containing these types of amino acids present higher proteolytic stability, improved pharmacokinetics, and increased membrane permeability, given the higher lipophilicity [141]. 

**Figure 10:**
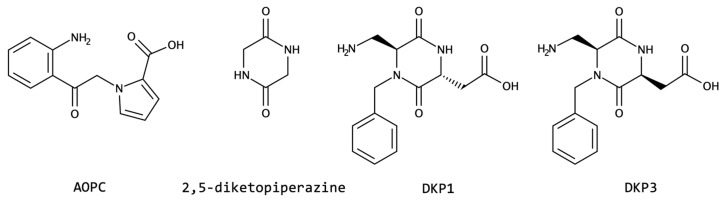
Selected examples of *N*-cyclized scaffolds resembling amino acids: AOPC, 2,5-diketopiperazine, DKP1, and DKP3.

Regarding unusual *N*-cyclization derivatives, in 2019, the Vassiliki Magafa group [142] developed new stable analogues of *Neurotensin* (NT; pGlu–Leu–Tyr–Glu–Asn–Lys–Pro–Arg–Arg–Pro–Tyr–Ile–Leu) containing non-canonical amino acids. The NT analogues with the residue AOPC (Figure 10), at position 8 of the peptide, are the ones with the best performance regarding enzymatic stability and binding to NT receptor 1. This peptide plays a dual role as a neurotransmitter/neuromodulator in the central nervous system and as a hormone/cellular mediator in the periphery [142]. 

Diketopiperazine-based (DKP) amino acids are other examples of modifications via cyclization at the main chain *N*. This scaffold was used to design both linear and cyclic CPPs containing DKP1 and DKP3 moieties (Figure 10), and computational as well as experimental conformational studies revealed well-defined helical structures in a micellar medium for the non-cyclic peptides, while cyclic peptidomimetics were more flexible [143]. Furthermore, the cyclic ones were particularly resistant to proteolytic degradation when compared with linear peptide chains and are, therefore, reliable templates for the design and biological modulation of new peptide therapeutics, including peptide carriers. Biological investigations showed higher membrane activity of cyclic derivatives, allowing their use as shuttles for anticancer drugs. The DKP moiety was also used to enhance integrin binding and tumor cell uptake, via a DKP-RDG ligand for prospective use in imaging and drug delivery [144].

#### 2.1.7. Other Side-Chain Modified Amino Acids 

**Figure 11:**
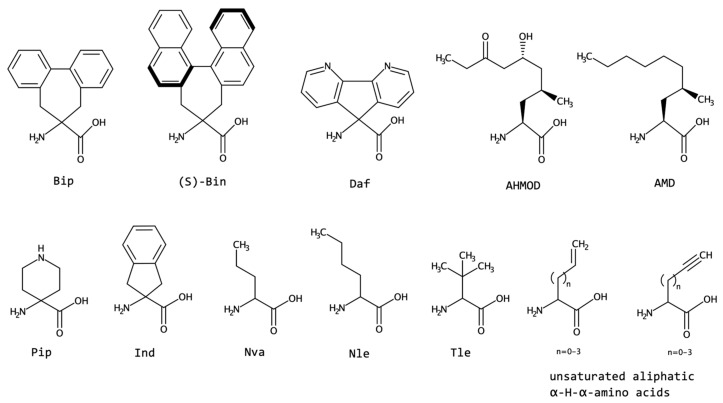
Selected examples of non-canonical amino acids: Bip (2′,1′:1,2;1″,2″:3,4-dibenzcyclohepta-1,3-diene-6-amino-6-carboxylic acid), Bin (1,1′-binaphthyl-substituted α-aminoisobutyric acid), Daf (9-amino-4,5-diazafluorene-9-carboxylicacid), AHMOD ((2S)-amino-(6R)-hydroxy-(4S)-methyl-8-oxodeca-noic acid), and AMD ((2S)-amino-(4S)-methyldecanoic acid). Pip (4-aminopiperidine-4-carboxylic acid), Ind (aminoindane carboxylic acid), Nva (norvaline or 2-Aminopentanoic acid), Nle (norleucine or (2*S*)-2-aminohexanoic acid), Tle (*tert*-leucine or *tert*-butylglycine), and the unsaturated aliphatic amino acids.

The amino acids and applications already mentioned show that ncAA have acquired considerable importance in the design of bioactive peptidomimetics. Figure 11 shows selected examples of ncAA residues that differ from the classes addressed above.

The amino acids Bin and Bip (1,1′-binaphthyl-substituted α-aminoisobutyric acid and 2′,1′:1,2;1″,2″:3,4-dibenzcyclohepta-1,3-diene-6-amino-6-carboxylic acid, respectively) are reported to combine structural features of both Db_z_g and Ac_7_c residues [145,146,147]. In fact, these ncAAs can be considered turn/helix inducers, and since they are rigid structures, they diminish peptides’ physiological vulnerability by making them difficult to access by proteases or peptidases [145,146,147,148]. Additionally, the ‘Bip method’ refers to the application of this amino acid as a probe for circular dichroism techniques [149].

Daf (9-Amino-4,5-diazafluorene-9-carboxylic acid) is another example of a rigid amino acid that imposes geometrical constraints when inserted into a peptide. This residue possesses the unique property of also being a ligand that can coordinate metal atoms. This fact is very important, allowing a broad spectrum of applications: metal-binding sites on proteins, peptide-based electronic devices, and molecular switches [150,151]. The expected conformations for Daf would be β-bends and α/3_10_-helix forms, since this residue can be classified as an α,α-disubstituted glycine, similar to Aib or Ac_7_c. However, a C5 conformation (fully extended form) was characterized experimentally, with a tendency to form a helical structure [150].

AHMOD ((2S)-amino-(6R)-hydroxy-(4S)-methyl-8-oxodeca-noic acid) and AMD ((2S)-amino-(4S)-methyldecanoic acid) are ncAA naturally found on *culicinin* peptaibols. Culicinins are peptides isolated from the fungus *Culicinomyces clavisporus* [152]. Importantly, *culicinin D* was found to exhibit potent antitumor activity [152,153]. The spatial structure of Culicinins is a right-handed helix, with a tighter *N*-terminus, forming a 3_10_-helix conformation [152]. The helical propensity of these residues should reflect the fact that these peptides also carry Aib. 

The non-canonical amino acids norvaline (Nva), norleucine (Nle), and *tert*-leucine (Tle) are hydrophobic residues. Nva and Nle proved to be helical-stabilizing amino acids [154,155]. Nva and Nle are found in small amounts in some bacterial strains [156]. Nva is known to promote tissue regeneration and muscle growth [157], while Nle can act as a methionine isostere [158]. In contrast, Tle does not induce the same constraint observed for Nva and Nle, varying with the environment and amino acid content of the peptide in which is inserted [159,160]. 

Pip (4-aminopiperidine-4-carboxylic acid) is a naturally occurring amino acid found on *Efrapeptin* peptides, which are produced by fungi of the species *Tolypocladium* [161]. This class of peptides has antifungal, insecticidal, and mitochondrial ATPase inhibitory activities [161,162]. The right-handed α-helical structure cannot be adopted by Pip-rich peptides. For *Efrapeptin*, the dominant structure is a 3_10_-helix [161]. Pip was also reported to increase the water solubility of peptides [159,163]. The non-canonical residue Ind (aminoindane carboxylic acid) has a stabilizing effect on the formation of α/3_10_-helices [159,164,165]. 

Another review by Rogers and Suga shows that genetic code reprogramming methods can generate functional peptides containing diverse non-canonical amino acids [166]. Selected examples of ncAA/modifications incorporated are Phe-like residues, Lys-like, peptoids, d-stereochemistry, and *N*-alkylated polycyclic.

Unsaturated aliphatic amino acids or olefinic amino acids occur in nature, especially in mushrooms, and have several bioactive roles. Vinylglycine, for instance, is an irreversible inhibitor of a variety of enzymes, and (S)-ethynylglycine possesses antibiotic activity [167]. Structurally, β-turn and β-hairpin were mentioned, but more relevance was given to their biological applications. 

Lastly, the incorporation of a fluorine-containing motif in an amino acid side-chain functions as a modulator of lipophilicity and solubility [168]. In fact, the -CF_3_ moiety is known to be active, as a wide number of commercial drugs incorporate this fluoroalkyl group [169,170]. Therefore, the inclusion of the trifluoromethyl group in peptides has risen in the last years, and the addition of this group to Cys, His, Trp, and Tyr has been successfully achieved. It preserved the native peptide structure and improved the metabolic stability, bioavailability, and cellular membrane permeability [171].

### 2.2. Backbone Modifications

The peptide backbone plays an important role in peptide stabilization. Modifications on the peptide backbone are another approach to generating peptidomimetics that are more conformationally constrained and thus more stable. Many types of backbone modifications have been performed and tested [6,11,12,50,172,173]. Very recently, strategies such as backbone extension, retro-inverso design, and bioisosterism were found to enhance the metabolic stability of a radiopharmaceutical peptide [174].

**Figure 12:**
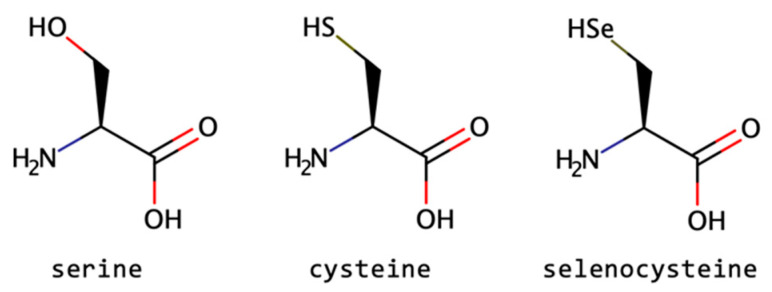
Example of three isoelectronic amino acids: serine, cysteine, and selenocysteine.

Basically, a backbone can suffer alteration by isosteric or isoelectronic substitutions, resulting in several types of mimetics. The isosteric modification consists in maintaining the same number of valence electrons but can differ in the number and type of covalently bound atoms: for example, N_2_ and CO, N_2_O and CO_2_, and N_3_^−^ and NCO^−^. Meanwhile, an isoelectronic substitution refers to two atoms, ions, or molecules sharing the same electronic structure and/or the same number of valence electrons but also the same structure (number of atoms and connectivity), as demonstrated in Figure 12 for serine, cysteine, and selenocysteine [6,10,12,173,175]. The bioisosterism strategy is used to reduce toxicity, change bioavailability, or modify the activity of a lead compound and may alter the metabolism of the lead. 

**Figure 13:**
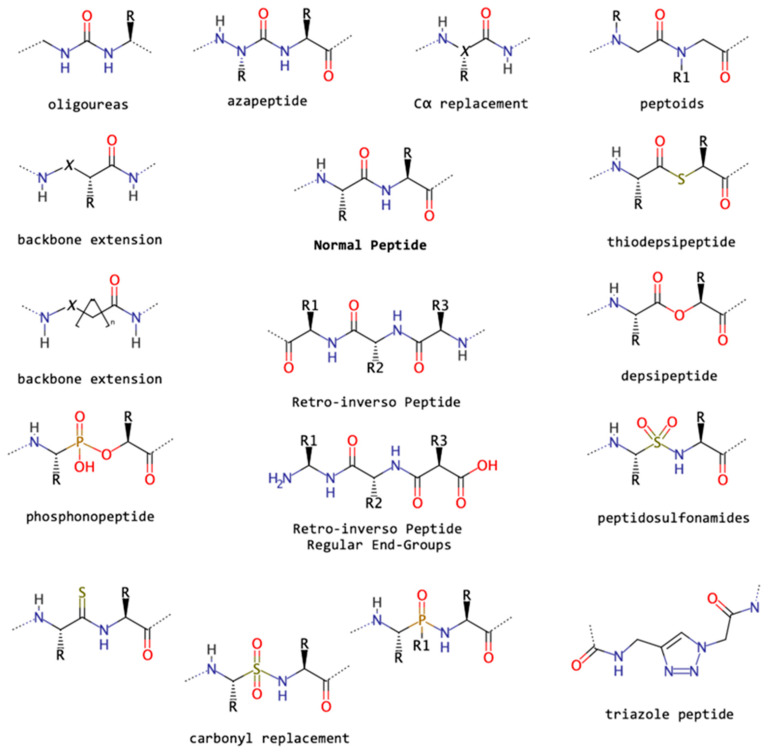
The two-dimensional structure of a natural peptide (center) surrounded by structures of known types of peptide backbone modifications.

Figure 13 summarizes the most important peptide backbone modifications: for instance, the replacement of Cα, backbone extension, and carbonyl replacement. In detail, we have azapeptides, in which an N atom replaces isoelectronically the Cα, yielding peptides that may be therapeutically applied as inhibitors of cysteine proteases [11,176,177]. Azapeptides presented as β-turn conformations, due to the lone-pair–lone-pair repulsion of the adjacent hydrazide nitrogen atoms [177].

Depsipeptides are relevant and active against several cancers [178,179,180]. Typically cyclic, they are the result of the replacement of an amide with an ester bond; in a peptide main chain, amide and ester bonds alternate. This modification has aroused great interest, since peptide esters have a lower propensity for intramolecular hydrogen bonding and therefore quite different molecular structure, which arises from the cyclic structure that confers proteolytic resistance, in place of the typical amide’s hydrogen bonds [181]. Thus, it is a target of investigation both computationally and experimentally, as pointed out by Thakkar and Engh and references therein [182]. Remarkable examples are the depsipeptides extracted from marine invertebrates, *Didemnin B*, *Plitidepsin* (dehydrodidemnin B) [183], and *Kahalalide F* [179]. In addition, *Romidepsin*, from a bacterium source, has shown relevant anticancer activity [178].

*Didemnin B* has remarkable biological activity, showing strong antiviral effect through the inhibition of the DNA and RNA synthesis; moreover, this peptide was one of the first to enter clinical trials to treat small-cell lung cancer and prostatic cancer [184,185,186]. *Plitidepsin* is a depsipeptide that carries a β-hydroxy-γ-amino acid, another example of a non-canonical residue. This peptide presents potent activity against antimyeloma in vitro and in vivo [187]. 

Retro-inverso peptides are generated when the amino acid sequence is reversed and the α-center chirality of the amino acid subunits is inverted as well, substituting the l-amino acids with their D forms. The use of these peptides is another approach to designing peptidomimetics that are more resistant to proteolytic degradation, but it does not always increase the pharmacological potency [11,188,189]. 

Retro-inverso peptides with regular terminal groups are able to either link to native peptides or be embedded in a large peptide generating potent peptidomimetics. One example of this is the peptide Tuftsin, which in its normal state is completely degraded in vivo in about 8 min. However, in retro-inverso peptide form, only 2% of hydrolysis is observed after 50 min, with the retention of its bioactivity [190].

A recent review reported novel applications for retro-inverso peptides, from immunology to antimicrobials [191]. Structurally, extended conformations will be less affected by the inversion, and the side chains will be well accommodated. However, for folded conformations, the maintenance of the original conformation can be more challenging and some strategies for ending groups, for instance, must be adopted to better mimic helical conformations [192,193].

Another family, peptoids, are formed by *N*-substituted glycines and have been considered a relevant motif for the design of novel molecules for decades [194,195]. This type of oligomer may result in stable synthetic polymers that also conserve natural biopolymers’ structure and function. Furthermore, peptoids will present properties consistent with the above explained *N*-alkylated ncAA (2.1.6). A comprehensive series of functionalized oligomers can be generated by adding groups to N. Peptoids are not able to form hydrogen bonds, as they lack the amide proton. Furthermore, the glycine core has no handedness and lacks a chiral center. These characteristics bring distinct conformational features in comparison to peptides. However, peptoids can be arranged in helical secondary structures, being applied in the design of antimicrobial peptides [196]. Simulation studies proved the application of peptoids in the design of nanosheets, where the peptoids adopt a linear backbone organization [197]. This type of *N*-substituted peptidomimetic has gained attention in both experimental and theoretical fields, being studied for multiple applications, such as drug carriers, cancer treatments, antibiotics, and antimicrobials [198,199].

Triazole-based peptidomimetics (Figure 13) have also emerged as interesting peptide-like compounds for several reasons, such as easy synthesis, conformational flexibility, and bioactive profile [200,201]. The small units 4Tzl and 5Tzl (1,4- and 1,5-substituted 1,2,3-triazole peptides, respectively) were addressed computationally via quantum chemistry calculations to predict their structural properties, and four different theoretical methods were compared regarding their robustness in describing these systems and helpfulness in the design of novel peptidomimetics [200].

Lastly, the backbone extension generates so-called β-amino acids (when there is an additional methylene between the amine and the acid), which constitutes a powerful foldamer strategy, generating unique helices, especially when cyclic or sugar-derivative side chains are present. However, γ-amino acids also display versatility and helical propensity, as reported by Martinek and Fulop [202], who also reviewed the structural properties of oligoureas and azapeptides (Figure 13) in the formation of stable helices. In addition, these authors characterized the combination of α, β, and γ amino acids in helical formation according to their explored dihedrals. This last strategy was also used to generate Endomorphin-2 peptidomimetics by replacing the Phe with the homologues β-hPhe. MD simulations and docking were performed to fully address the structural properties of the mimetics and their impact on inhibitory activity [203].

## 3. Conclusions

This review focused on four important topics: the difference between canonical and non-canonical amino acids, the relation between peptide secondary structure and biological function, the most relevant non-canonical amino acid classes, and the most common peptide backbone structure modifications. 

Table 1 summarizes the conformational preferences of the non-canonical amino acids that stand out within their class, illustrating different ways to generate peptidomimetics. 

Incorporating non-canonical amino acids into known peptides proves that this is a feasible and simple path to optimize the characteristics of native peptides, improving their activity and stability. 

A few more examples of peptidomimetics incorporating the reviewed ncAAs that are applied in very distinct processes and diseases are as follows: *Saralasin*, an angiotensin II analogue, has been effectively employed in the treatment of hypertension. Its sequence incorporates sarcosine at position 1, enhancing resistance to degradation by aminopeptidase and resulting in improved bioactivity of the compound [18,204]. *Icatibant* is a competitive antagonist of the bradykinin 2 receptor, utilized for the treatment of acute attacks of hereditary angioedema in patients with C1-esterase inhibitor deficiency. For this therapeutic peptidomimetic, the resistance to degradation is achieved through the incorporation of non-natural amino acids, such as hydroxyproline, l-2-thienylalanine, tetrahydroisoquinolinecarboxylic acid, and octahydroindolecarboxylic acid [205]. Lastly, *Carbetocin* is a peptidomimetic consisting of a cyclic eight-amino-acid sequence derived from oxytocin. It is employed to effectively manage postpartum hemorrhage, especially during caesarean sections, by targeting peripheral oxytocin receptors. Notably, *carbetocin* incorporates methyltyrosine at position 2 and replaces the disulfide bond with a more stable thioether linkage. This modification significantly enhances the compound’s metabolic stability compared to previous generations of lead compounds [206].

Regarding the backbone modifications strategy, we summarized here some mimetics with relevant biological functions, namely the azapeptides, depsipeptides, retro-inverso peptides, peptoids, and χ or β-amino acids, as shown in Table 2. 

We believe that peptidomimetics have the potential for a large variety of applications for biodevices, biosensors, and biomaterials able to capture specific interactions with physiological environments relevant in several fields, such as medicinal chemistry and biotechnology.

## Figures and Tables

**Table 1:** Conformational preferences and applications of the non-canonical amino acids addressed in this study.

ncAA Class	Highlights	Conformational Preferences	Characteristics	Application
symmetric α,α-dialkyl glycines	Aib	3_10_-helix or α-helix	increased proteolytic resistance helical foldamers	antimicrobial/antibiotic peptidomimetics
	Dhg	α-helix		
asymmetric α,α-dialkyl glycines (d-amino acids)	Iva, MDL and MDP	3_10_-helix or α-helix	increased proteolytic resistance helical foldamers	antimicrobial/antibiotic peptidomimetics
Cα to Cα cyclized (Ac_n_c residues)	Ac_3_c	bridge region	foldamers	neurotransmitters antimicrobial/antibiotic peptidomimetics
	Ac_6_c	3_10_-helix or α-helix		
	(*R,R*)Ac_5_c^dOM^	3_10_-helix or α-helix		
proline analogues	Hyp	β-turn, bend	foldamers	antimicrobial peptidomimetics
β-substituted amino acids	β-MePhe Tmt, Tic	Side-chain constraint	increased proteolytic resistance hormones mimetics	antinociceptive activity (opioids)
α,β-dehydroamino acids	Δ^z^Phe ΔAbu	β-turn or γ-turn 3_10_-helix or α-helix	increased proteolytic resistance hydrogels	drug delivery cancer treatment
*N*-alkylated	sarcosine	cyclic peptides	increased lipophilicity improved pharmacokinetics	antibiotic immunosuppressant
*N*-cyclization	DKP1 DKP3	helix	increased proteolytic resistance	neurotransmitter neuromodulator drug delivery anticancer
other	Bip Bin	turn/helix inducers	increased proteolytic resistance	circular dichroism probe
other	(S)-Ethynylglycine	β-turn β-hairpin	foldamers	antibiotic activity

**Table 2:** Biological application and preferable secondary structure of peptidomimetics based on backbone modifications.

Backbone Modification	Highlights	Conformational Preferences	Characteristics	Application
azapeptides	Ac-l-Phe-azaAlaOiB Ac-l-Phe-azaGlyOMe Boc-(Phe-azaPhe-Ala)_2_-OMe)	β-turn extended	increased proteolytic stability	inhibitors of cysteine proteases
depsipeptides	*Didemnin B* *Plitidepsin* *Kahalalide F* *Romidepsin*	cyclic	increased flexibility	antiviral cancers treatments
retro-inverso	*Amytrap* BMAP-28_D_(LPR); _D_(RGD)	extended helix	resistant to proteolytic degradation	anticancer immunology neurodegenerative diseases antimicrobial diagnosis
**peptoids**	Triazole-peptidomimetics	helix sheets	stable synthetic polymers	antimicrobial drug carrier anticancer antibiotics

## Data Availability

Data from cited papers written by us are available upon request.
